# Oral Neutrophil Free Fatty Acid Receptors Expression May Link Oral Host and Microbiome Lipid Metabolism

**DOI:** 10.3389/froh.2022.821326

**Published:** 2022-03-07

**Authors:** Martin Wzatek, Shaima Bahammam, Petronela Buiga, Kendal Haddad, Corneliu Sima

**Affiliations:** ^1^Department of Oral Medicine, Infection, and Immunity, Harvard School of Dental Medicine, Boston, MA, United States; ^2^Department of Dentistry, Faculty of Medicine, Masaryk University, Brno, Czechia; ^3^Department of Dentistry, King Faisal Specialist Hospital and Research Center, Riyadh, Saudi Arabia

**Keywords:** oral neutrophils, free fatty acid receptor, FFAR, ERV1, fish intake

## Abstract

In health, commensal bacteria from oral biofilms stimulate polymorphonuclear neutrophil (PMN) recruitment in gingival sulci and the oral cavity. Oral PMN (oPMN) is short-lived cells with low prosurvival gene expression. In periodontitis, oPMN accumulates in higher numbers, has extended lifespan, and sustains nonresolving inflammation. We hypothesize that short- and long-chain free fatty acids (SCFAs and LCFAs) and lipid mediator resolvin E1 (RvE1) modulate host ability to control biofilms and resolve inflammation. Our objective was to measure oPMN surface expression of receptors FFAR2 (binds bacteria-derived SCFA), FFAR4 (binds LCFA, EPA, and DHA), and ERV1 (binds RvE1) in health and to assess sex differences. We included 20 periodontally healthy individuals aged 20–80 years (10 males, 10 females), who were asked to (1) answer a targeted health nutritional questionnaire and (2) provide an oral saline rinse. oPMN isolated by sequential filtration was labeled with fluorophore-conjugated antibodies against CD11b, CD14, CD16, CD66b, ERV1, FFAR2, and FFAR4 and analyzed by flow cytometry. Statistical analyses were the following: two-way ANOVA, Tukey's test, and Pearson's correlation. Oral rinses contained 80% oPMN of which 60% were ERV1^+^ and FFAR2^+^, and 10% FFAR4^+^, with no sex differences. Females had more oPMN ERV1 compared to males. Both sexes had higher ERV1 compared to FFAR2 and FFAR4. CD66b^+^CD16^high^ oPMN expressed less ERV1 and FFAR2 compared to CD66b^+^CD16^low^. There were positive correlations between oPMN ERV1 and FFAR2 expression and between ERV1^+^ and FFAR2^+^ oPMN and fish intake. These findings will help to better understand how oral host and microbiome interactions maintain periodontal health.

## Introduction

In health, commensal bacteria in the gingival biofilm stimulate the recruitment of polymorphonuclear neutrophils (PMN) in the periodontium, gingival crevicular fluid, and the oral cavity. Oral PMN (oPMN) are short-lived cells with low prosurvival gene expression. In periodontitis, oPMN accumulate in higher numbers, have extended lifespan, and sustain nonresolving inflammation. It is believed that, like intestinal bacteria, oral bacteria associated with periodontal health induce immune tolerance and prevent the host immune system from being overactivated. It is also likely that noninvading commensal bacteria in the gingival sulcus maintain the continuous influx of PMN that contribute to control of subgingival biofilm composition without collateral tissue damage [1, 2]. Short-chain fatty acids produced by the gut and oral microbiomes, and binding to free fatty acid receptor 2 (FFAR2) in leukocytes, may be positive regulators of the host-biofilm balance [3, 4]. The shift from inoffensive to pathogenic subgingival biofilms remains poorly understood but always associates with nonresolving periodontal inflammation and loss of bone around teeth.

Omega-3 (Ω-3) binding to FFAR4 and derived specialized proresolving lipid mediators (SPMs), including resolvin E1 (RvE1) binding to its receptor ERV1, play important roles in the resolution of inflammation [5, 6]. It was recently reported that females are more efficient in resolving inflammation compared to males [7]. We therefore aim to determine the normal levels, sex differences, and healthy vs. periodontitis differences in oPMN receptors and lipid ligands of inflammation resolution in the oral cavity. Further understanding the sex differences and oPMN changes in patients with periodontitis will help in mapping the host's innate immune control over the oral biofilm to prevent its pathogenic shift that associates with periodontitis. This is important because periodontitis is a highly prevalent chronic inflammatory disease characterized by progressive loss of tooth-supporting structures in almost 50% of US adults [8]. Severe periodontitis affects 10–15% of the world population causing significant deterioration of oral health-related quality of life [9–11]. Further, oral microbiome dysbiosis and chronic inflammation seen in periodontitis have been associated with systemic conditions including diabetes and cardiovascular diseases among others [12–14].

The central hypothesis is that free fatty acids and derived active mediators (e.g., RvE1) produced by the host and microbiome modulate hosts' ability to control biofilm pathogenicity and resolve inflammation, through FFAR2, FFAR4, and ERV1. The aim of this pilot study was to measure oPMN surface expression of receptors FFAR2 (binds bacteria-derived SCFA), FFAR4 (binds LCFA, EPA, and DHA), and ERV1 (binds RvE1) in health and to assess sex differences in their expression. The secondary aim was to assess correlations among oPMN receptors, fish intake, and body mass index.

## Methods

### Subjects

**Figure 1:**
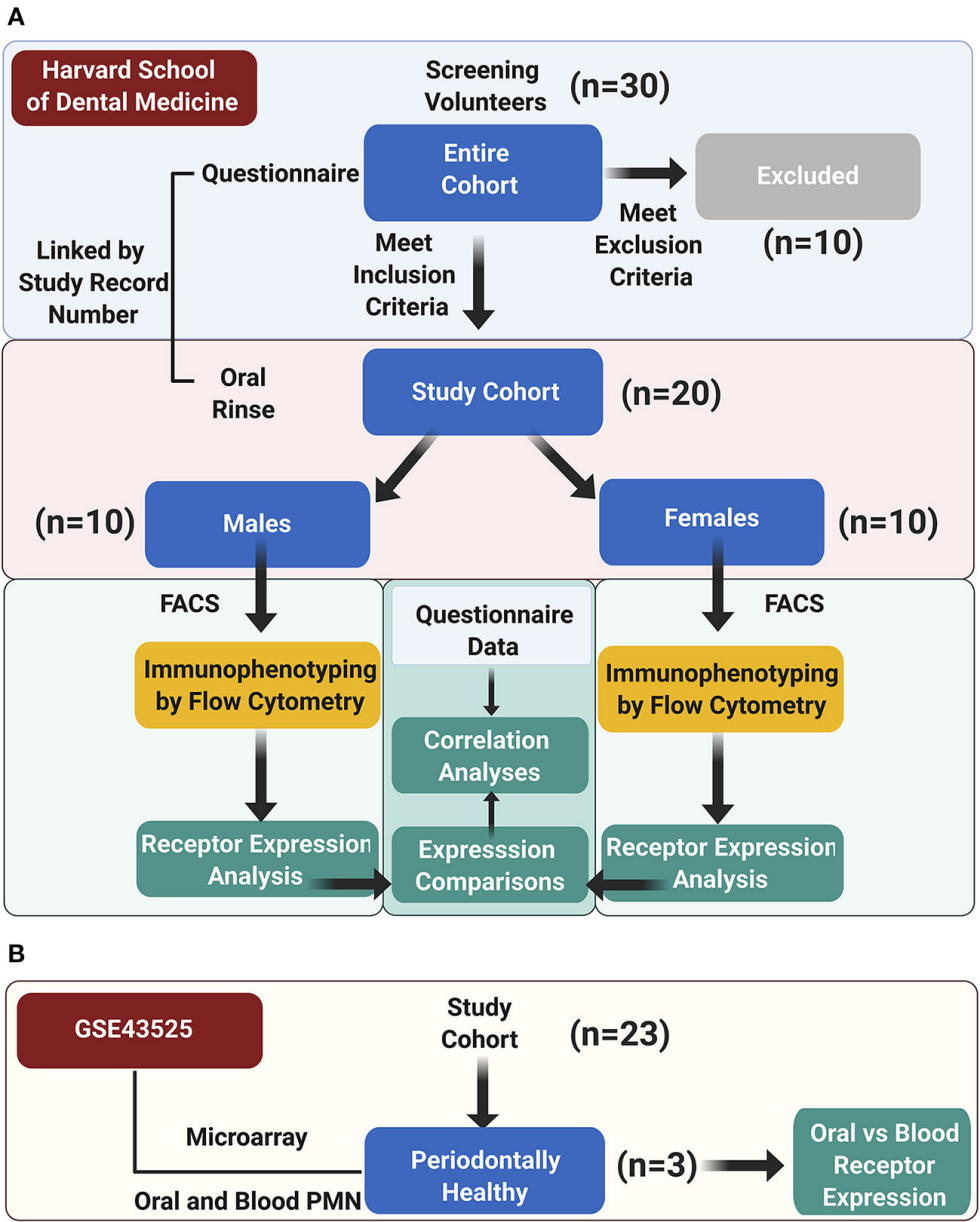
Study design. **(A)** The study cohort enrolment, data acquisition, and analysis workflow at Harvard School of Dental Medicine are illustrated; **(B)** an auxiliary microarray data analysis was performed on the GSE43525 dataset.

Study subjects were screened at Harvard School of Dental medicine from patients, students, residents, and faculty by verbal self-reporting of oral health, minimum of 20 teeth present, no acute conditions (e.g., bacterial, viral, or fungal acute infections), no chronic oral mucosal conditions (e.g., mucous membrane pemphigoid, erosive lichen planus, pemphigus), and no long-term antiinflammatory or antibiotic medications, and not having eaten, brushed or used a mouth rinse in the past 2 h before sample collection. A total of 20 periodontally healthy individuals >20 years old (10 men, 10 women) were included (Figure 1A). Informed consent to participate was obtained from all participants. The protocol was approved by the Institutional Review Board of the Harvard Longwood Medical Area (IRB19-1697). This work complies with the guiding principles for experimental procedures found in the Declaration of Helsinki of the World Medical Association.

### oPMN Isolation

All oral rinse samples were collected at least 2 h after brushing or eating to avoid hygiene and dietary interference with the test results. Participants were asked to rinse three times: the first rinse (5 ml of tap water) was used for the oPMN count, the second and third rinses (15 ml of saline water per rinse) were used for the isolation of oPMN and their analysis by flow cytometry. A minimum of 2 min was counted between the three rinse samples.

### Flow Cytometry

**Figure 2:**
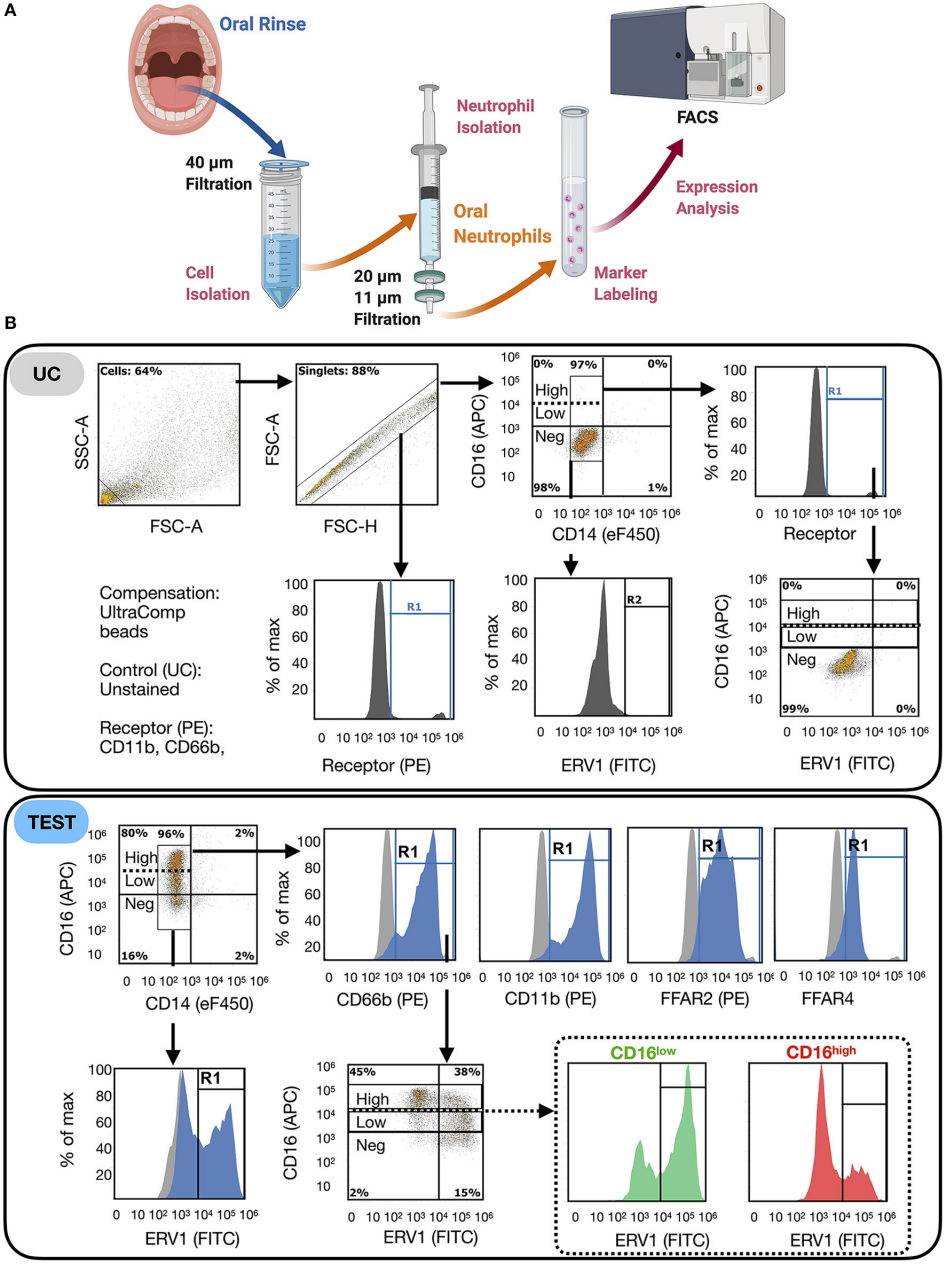
FACS protocol for oPMN immunophenotyping. **(A)** oPMN were isolated from rinses by sequential filtration (20 min), labeled (1 h) and analyzed by fluorescent-activated cell sorting (FACS) for receptor expression. oPMN: identified as CD14^−^CD16^+^CD66b^+^CD11b^+^; **(B)** gating strategy: debris was excluded on SSC-A vs. FSC-A and doublets were excluded on FSC-A vs. FSC-W; true positivity was determined using unstained control (UC, >98% negative on each channel) for each sample; percentage cells positive for oPMN markers, CD11b and CD66b were calculated on singlets, which were then plotted on CD16 vs. CD14 to exclude monocytic myeloid cells (CD14^+^); the percentage of cells positive for each receptor and the mean fluoresce intensity were measured on oPMN and compared between groups (males vs. females); ERV1 and FFAR2 were measured in CD16^low^ and CD16^high^ oPMN populations.

All samples were collected bench-side to minimize the time from collection to analysis. Rinse samples were immediately transferred on ice and processed for flow cytometric analysis, which was run within 2 h of collection to minimize cell activation and cell death. Since most receptors included in the analysis are stored in cytoplasmic granules before expression on the cell surface, and the purpose of the study was to measure surface expression, cells were run fresh and not fixed. Fixation can increase granulocyte permeability and result in staining of both intracellular and surface antigen epitopes [15–18]. oPMN was isolated from oral rinse samples (rinse two and three) by sequential filtering through 40-, 20-, and 11-μm filters, kept on ice (Figure 2A). Cell viability was determined by Trypan blue exclusion on a hemocytometer (>80% viability across the sample set).

Spun down samples were concentrated to one million cells per ml and labeled for the markers of interest. Nonspecific binding was blocked with 1% BSA and 50 μg/ml human IgG (Invitrogen) and oPMN labeled with 1 μl of each fluorophore-conjugated antibodies against CD14 (eFluor450), CD16 (APC), ERV1 (FITC), CD11b, CD66b, FFAR2, and FFAR4 (PE) (eBioscience/ThermoFisher) were analyzed on AttuneNxT BRV6 within 2 h of collection. Calibration was performed with antibodies conjugated to magnetic beads (UltraComp eBeads, eBioscience) and voltage equivalency among experiments established using peak five rainbow beads (Spherotech). Each sample was run in duplicate, and data were analyzed using the Attune NxT Software v3.1 and FlowJo v10.

### Questionnaire

A study questionnaire was used to collect relevant medical and dental information. Subjects were asked for self-reported oral and medical diagnoses, BMI, medications including baby aspirin, ω-3 supplements, weekly fish intake, and pregnancy trimester for pregnant women. All subjects included in the study had been seeing their primary care physician and dentist at least yearly for the past 5 years, which increases the reliability of self-reporting of oral and systemic health status. Food frequency questionnaires including recall of average weekly fish servings per week previously validated with biomarkers were found to correlate strongly with blood ω-3 FA levels and have been used in epidemiological studies, including the National Health and Nutrition Examination Surveys (NHANES) [19–24]. The correlations among receptors of interest, age, BMI, and fish intake/ω-3 supplements were investigated.

### Bioinformatic and Statistical Analyses

Secondary data analysis for differential gene expression (DEG) was performed for microarray data of periodontally healthy subjects from GSE43525 [2, 25]. In the original study, PMN were isolated from venous blood and oral rinse samples obtained from chronic periodontitis patients and healthy subjects, and gene expression microarray analysis was performed. In this study, only samples from periodontally healthy subjects (*n* = 3, one female and two males) were analyzed by comparing the gene expression changes in oral vs. blood PMN (Figure 1B). GEO2R, an R-based web application, was used to compare two groups of samples to identify genes that are differentially expressed across phenotypic changes in oPMN compared to blood PMN in healthy subjects [26]. *P*-Values were adjusted using Benjamini and Hochberg false discovery rate [27]. To assess the three proteins of interest FFAR2 (GPR43), FFAR4 (GPR120), and ERV1 (CMKLR1) in the context of their interacting network, these were further processed to “built network”, using the “analyze network” algorithm, one of the nine network-building algorithms in MetaCore. The *p*-values of the resulting network are the possibility of the potential networks according to the curated human protein interaction database within the MetaCore from Clarivate Analytics (https://portal.genego.com/cgi/data_manager.cgi). Groups were compared by two-way ANOVA, paired *t*-tests and Tukey's test, and correlations between normally distributed marker values by Pearson. Analyses were performed in GraphPad Prism v9.

## Results

To investigate the expression of free fatty acids receptors on oPMN, FFAR2, FFAR4, and ERV1 were quantified in cells isolated from oral rinses from periodontally healthy individuals. Isolated cells were immunophenotyped by flow cytometry as described in Methods. Equal numbers of males and females were included to assess sex differences in receptor expression and their relationships with age, BMI, and fish intake (Supplementary Table S1). All participants answered a questionnaire on relevant oral and systemic health and also medications, ω-3 supplements, and weekly fish intake (Supplementary Table S2). Only one study subject reported taking ω-3 supplements on a regular basis. Hence, most supplemented it from the diet: 70% of study participants reported consuming fish every week with 30% having on average <1 serving of fish per week (*n* = 6), 25% (*n* = 5) having one serving per week, and 45% (*n* = 9) having two or more servings per week. There were no significant differences between males and females for any variable.

**Figure 3:**
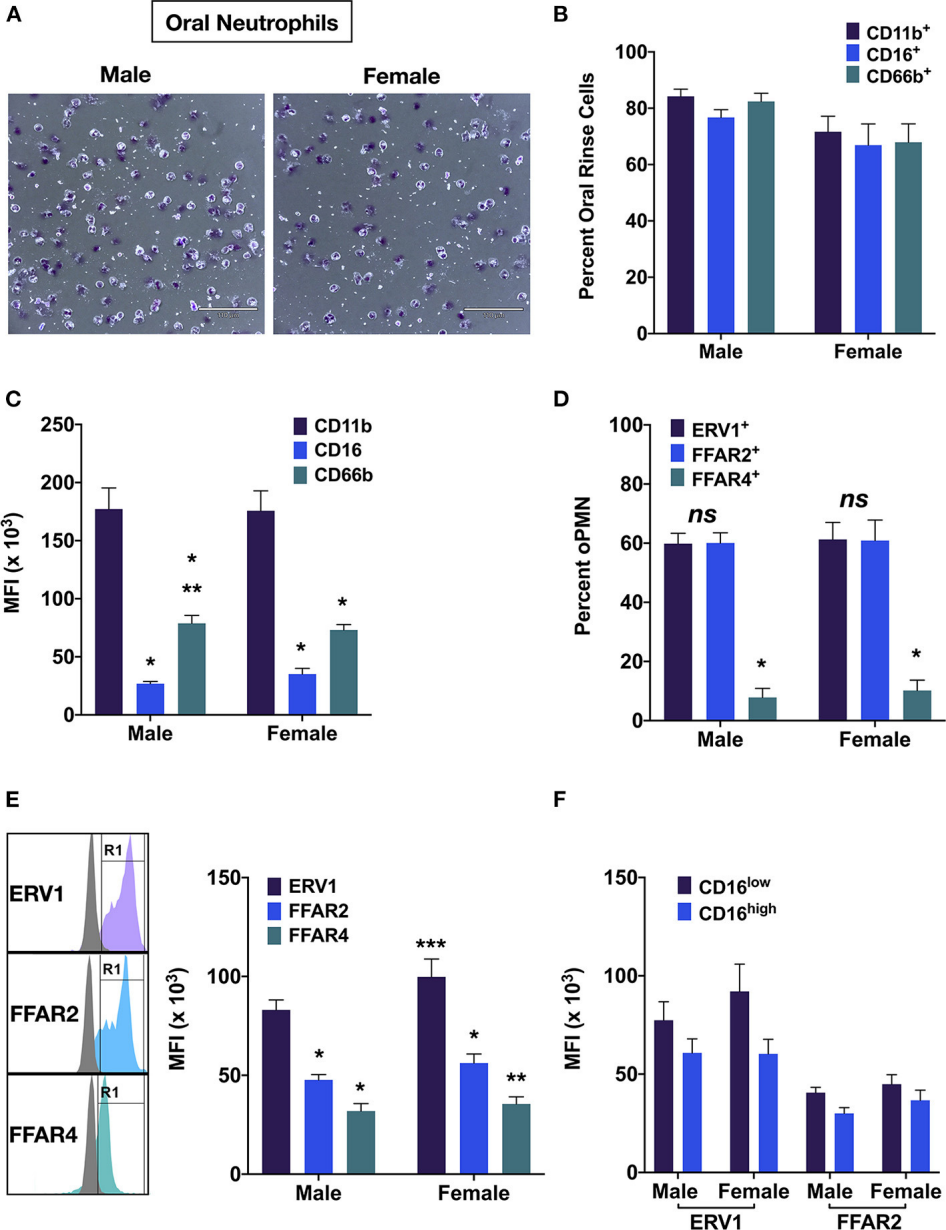
OPMN expression of free fatty acid receptors. **(A)** Representative images of male and female oPMN (Giemsa stain). **(B)** Percentage of oral rinse cells and positive for CD11b, CD16, and CD66b, and **(C)** their expression by oPMN (Tukey's test: *p* < 0.01, * vs. CD11b; ** vs. CD16). **(D)** Percentage ERV1^+^, FFAR2^+^, FFAR4^+^ oPMN and **(E)** expression levels (Tukey's test: **p < 0.05 FFAR2 vs. FFAR4 *** females vs. males). **(F)** ERV1 and FFAR2 levels in CD16^high^ and CD16^low^ oPMN (*p* < 0.01 CD16^high^ vs. CD16^low^; ns males vs. females).

Flow cytometric analyses revealed that more than 90% of oral immune cells were myeloid (CD11b^+^), and >80% in males and >75% in females were oPMN (CD66b^+^) (Figures 2B, 3A,B). Male oPMN expressed more CD66b compared to female, and both had high CD11b expression compared to CD16 and CD66b (Figure 3C). On average, 60% of oPMN expressed ERV1 and FFAR2 in both males and females, and only 10% expressed FFAR4 (Figure 3D). ERV1 expression was highest (97 ± 6 × 10^3^ MFI) followed by FFAR2 (52 ± 3 × 10^3^ MFI) and FFAR4 (33 ± 22 × 10^3^ MFI) in both sexes (Figure 3E). To compare receptor expression levels between viable (CD16^high^) and apoptotic (CD16^low^) oPMN, the two distinct populations were assessed and CD16^low^ oPMN was found to express higher ERV1 and FFAR 2 levels compared to CD16^high^ (Figure 3F).

**Figure 4:**
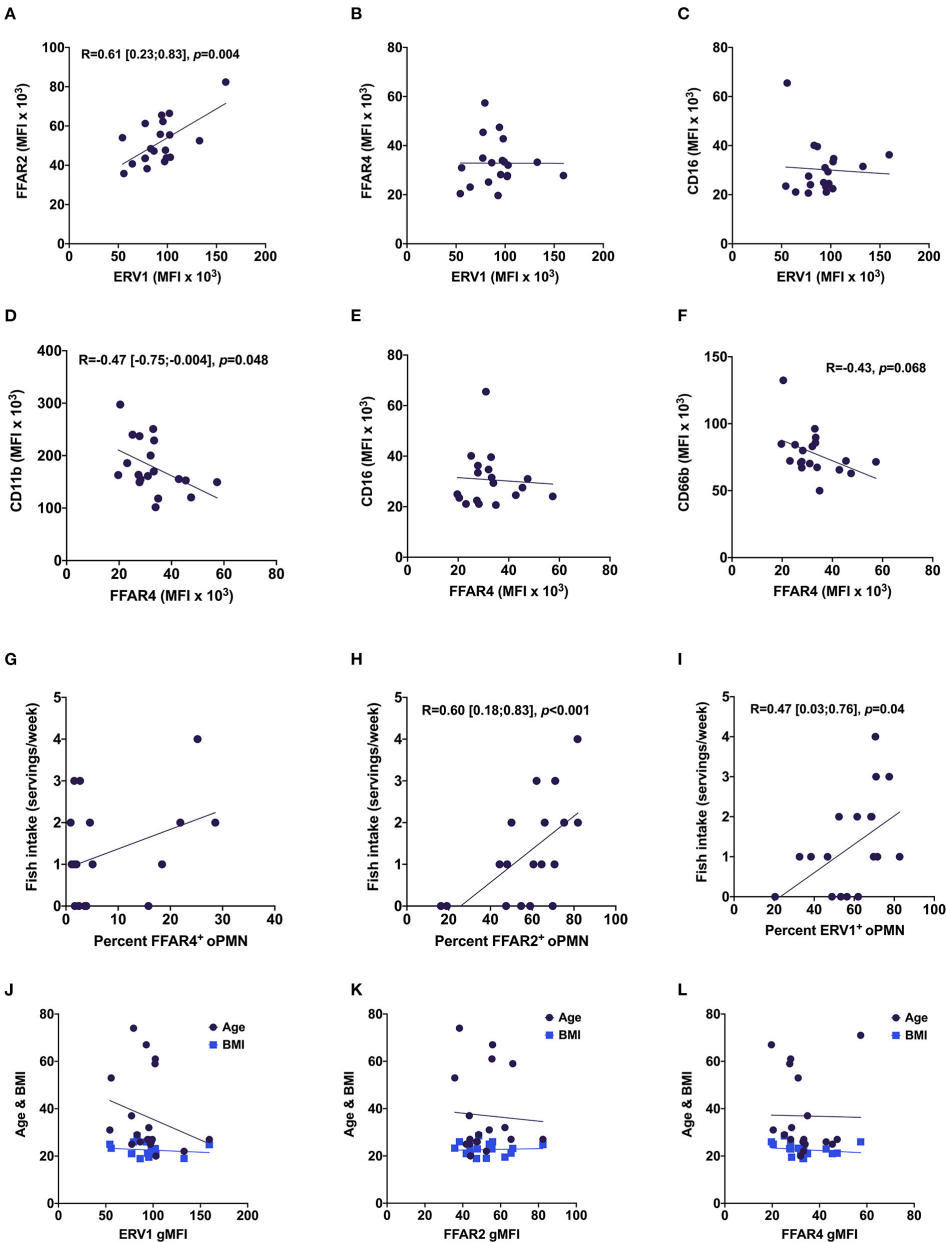
Relationships between oPMN free fatty acid receptors, oPMN activity markers, and fish intake. Correlations between ERV1 and FFAR2 **(A)**, FFAR4 **(B)**, CD16 **(C)**; between FFAR4 and CD11b **(D)**, CD16 **(E)** and CD66b **(F)**; and between FFAR4^+^ **(G)**, FFAR2^+^ **(H)** and ERV1^+^ **(I)** and fish intake, and age and BMI **(J–L)** (Pearson's, *p* < 0.05 for **A,D,H,I**).

We next interrogated the relationships among FFAR2, FFAR4, and ERV1 and also with age, BMI, and fish intake. There were high correlations between ERV1 and FFAR2 expression [*R* = 0.61, 95% CI (0.23, 0.83)], and between the percentage of FFAR2^+^ oPMN and fish intake [*R* = 0.60, 95% CI (0.18, 0.83)] (Figures 4A,H). Further, a moderate correlation was found between ERV1^+^ oPMN and fish intake [*R* = −0.47, 95% CI (0.03, 0.76)] and moderate inverse correlation between CD11b and FFAR4^+^ oPMN [*R* = 0.60, 95% CI (−0.75, −0.004)] (Figures 4D,I). No significant correlations were found between either free fatty acid receptor and CD16 or between FFAR4 and ERV1, FFAR2, or fish intake (Figures 4C,E–G). Similarly, no significant correlations were found between either receptor expression and age or BMI (Figures 4J–L).

**Figure 5:**
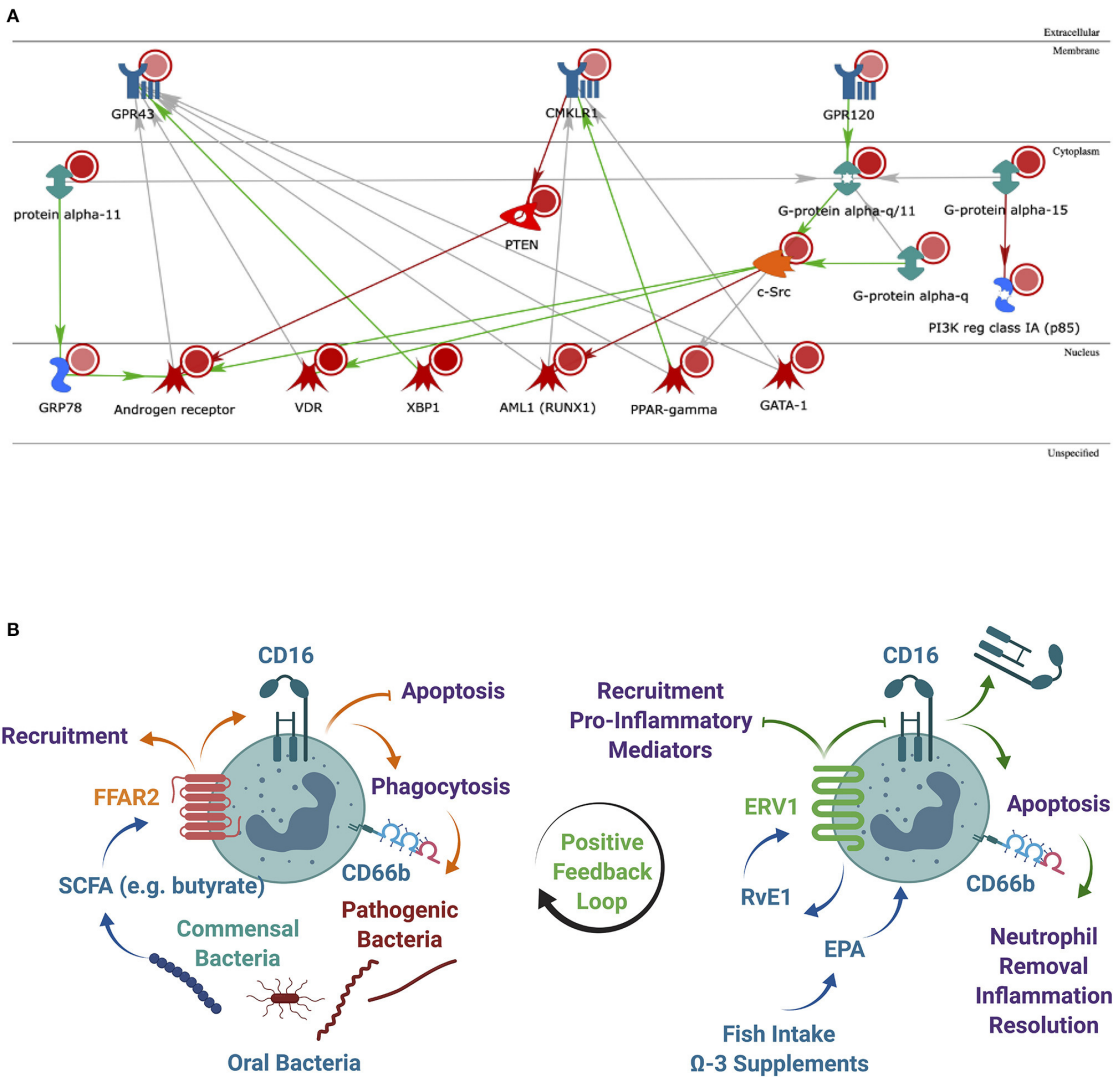
Analysis of the most significantly regulated networks. **(A)** Relationships among the regulated human proteins in healthy subjects when comparing oral vs. blood samples. Networks of protein interactions in regulated human proteins [including FFAR2 (GPR43), FFAR4 (GPR120), and ERV1 (CMKLR1)] predicted by MetaCore from Clarivate Analytics. Blue objects in the membrane represent receptors. Red circles–upregulated expression value. Green both-end arrows are G-alpha GTPases; PTEN, phosphatase and tensin homolog deleted on chromosome 10; orange object cytoplasm is protein kinase (c-Src). Blue objects generic binding proteins. Red objects in the nucleus represent transcription factors. Green arrows represent activation and red arrow-inhibition and gray arrow-unspecified; **(B)** a lipid metabolite positive feedback loop may exist between commensal oral bacteria *via* FFAR2 and inflammation resolution *via* ERV1. The expression of FFAR2 in oPMN to respond to SCFAs from commensal oral bacteria correlates with ERV1 expression to facilitate the effective control of pathogens and to resolve inflammation. SCFA, short-chain fatty acid; LCFA, long-chain fatty acid; RvE1, resolving E1.

We further examined the intracellular pathways linking the three receptors in immune cells by secondary analysis of a microarray dataset on RNA extracted from oPMN of three periodontally healthy individuals. Our previous findings on this full dataset identified the NRF2 antioxidant pathway downregulated in patients with periodontitis compared to health [2]. Using MetaCore from Clarivate Analytics, networks between the three receptors of interest–FFAR2 (GPR43), FFAR4 (GPR120), and ERV1 (CMKLR1) were calculated based on the “analyze network” algorithm value, and then, the network maps of their putative protein interactions and related proteins were predicated accordingly from the database. It was noted that, compared to blood PMN, oPMN have increased ERV1 expression, which inhibits the PTEN phosphatase that in turn inhibits the androgen receptor (AR). This contrasts with GPR120 (FFAR4) activating the AR through upregulation of c-Src (Figure 5A). Given the significantly higher ERV1 expression in these cells compared to FFAR4, it is reasonable to assume that the former effect prevails.

## Discussion

To the best of our knowledge, this is the first report on SCFA and LCFA receptors expression in oPMN and their relationships to fish intake and markers of cell activation. It is believed that, like intestinal bacteria, oral bacteria associated with periodontal health induce immune tolerance and prevent the host immune system from being activated. It is also likely that noninvading commensal bacteria in the gingival sulcus maintain the continuous influx of PMN that contributes to the control of subgingival biofilm composition without collateral tissue damage [1, 2]. SCFAs produced by the microbiota in the gut and oral microbiomes, and binding to FFAR2, may be positive regulators of the host-biofilm balance [6]. The shift from inoffensive to pathogenic subgingival biofilms remains poorly understood but always associates with nonresolving periodontal inflammation and loss of bone around teeth. Ω-3 binding to FFAR4 and derived SPMs, including RvE1 binding to ERV1, play important roles in the resolution of inflammation [3, 4]. Based on our corroborated findings and known functions of CD16 in PMN [7], we hypothesize that a positive feedback loop exists between expression of these two receptors and downstream actions to maintain oral homeostasis.

Metatranscriptomics of the human oral microbiome during health and disease revealed several metabolic pathways upregulated in periodontitis vs. health, including lysine degradation to SCFA butyrate predominantly by *Fusobacterium nucleatum* [28]. On the contrary, a more recent metatranscriptomic study found reduced bacterial activity of the lysine pathway in periodontitis compared to health, which indicates a regulatory action of lysine-derived SCFA [29]. Further, SCFAs butyrate and propionate were found higher in gingival crevicular fluid of gingivitis patients compared to periodontally healthy individuals [28]. Mounting evidence from gut microbiome research indicates a beneficial regulatory action of SCFA for immune tolerance, inflammation resolution, and maintenance of gut homeostasis, in part through the inhibition of the activity of histone deacetylases [30–32]. Nonetheless, FFAR2-deficient mice (*Gpr43*^−/−^) showed exacerbated or unresolving inflammation in models of colitis, arthritis, and asthma and increased alveolar bone resorption [33, 34].

SCFAs such as butyrate produced by the digestive tract bacterial processing of dietary fiber reduces the development of inflammatory and metabolic disorders in humans and improved insulin sensitivity concurrent with increased energy expenditure in murine diet-induced obesity [33, 35]. Maslowski et al. showed that SCFAs induce a robust calcium flux in mouse and human PMN, but not in PMN from *Gpr43*^−/−^ mice, which indicates that GPR43 is the sole functional receptor for SCFAs on PMN [33]. Similarly, resolvin E1 (RvE1), a ligand for ERV1 (aka ChemR23, CMKLR1) in myeloid cells inhibited PMN recruitment, stimulated phagocytosis and inhibited production of proinflammatory mediators in murine models of inflammatory and metabolic diseases in part through S6 phosphorylation downstream of the PI3K/Akt and Raf/ERK pathways [36–38]. Importantly, RvE1 induces PMN apoptosis–a hallmark of inflammation resolution initiation, *via* caspases 3 and 8 and induction of mitochondrial disfunction by attenuating ERK and Akt-mediated apoptosis-suppressing signals [39]. When PMN undergo apoptosis, they lose expression of the surface receptor CD16 (Fcγ RIIIb). Thus, levels of surface CD16 are good indicators of apoptotic or nonapoptotic PMN [40]. We have previously found that in health, two distinct oPMN subpopulations exist, which can be differentiated by CD16, CD55, and CD63 surface expression [1]. This is consistent with the theory that the oral cavity is in a parainflammatory state, the intermediary immune state that allows the host to adequately respond to low-grade noxious agents or tissue damage, without clinical signs of inflammation [41, 42]. Therefore, the mechanisms that regulate oPMN function in health allowing for tolerance of the commensal microbiota and management of opportunistic pathogens while avoiding tissue damage may be essential regulators of the parainflammatory state (Figure 5B).

Our findings also show that ERV1 signaling in PMN can block the inhibitory actions of PTEN on AR expression, therefore indirectly potentiating its signaling. The AR is known to regulate both differentiation and function of PMN in males, with apparent beneficial actions for the host, to survive bacterial challenge [43, 44]. On the flip side, males are less prone to autoimmune diseases and appear to resolve inflammation less efficiently compared to females [7]. It has long been thought that the male sex hormones may modulate the development and function of immune cells. Males are at higher risk of developing sepsis, acute respiratory distress, and multiorgan failure after traumatic hemorrhagic shock and thermal injury, in part because of immune suppression and abnormal activation of PMN [45]. More recently, Chuang et al. demonstrated an essential role for the AR in granulopoiesis and host defense against microbial infection [44]. Notably, the major induction of Stat3 reporter activity in PMN was driven by AR expression and was only slightly enhanced by adding androgens [44]. This suggests that the function of AR in promoting Stat3 activity in PMN may be independent of androgens.

Limitations of this pilot study include (1) small sample size to represent all demographic groups, and thus, receptor expression findings cannot be extrapolated to the entire population, (2) lack of blood samples to measure ω-3 FA levels and clinical examinations for validation of the administered questionnaire, and (3) limited number of samples for bioinformatic analyses, which prevented oral vs blood comparisons between males and females. However, our findings helped to generate the hypothesis that a positive feedback loop may exist between oral host and microbiome metabolisms potentially mediated by dietary ω-3 FAs. This hypothesis will be tested on a larger cohort in a separate study that would also aim to confirm the current findings and get insights into how oPMN FFARs expression changes in subjects with periodontal diseases.

In conclusion, our findings show that the majority of human oPMN express the SCFA receptor FFAR2 (GPR43) and LCFA receptor ERV1, the latter being at higher levels in females compared to males. Significant positive correlations between FFAR2 and ERV1 and also between each receptor and fish intake (ω-3-rich) suggest a possible positive feedback loop mediated by oral bacteria signaling through oPMN to control microbiome pathogenicity, resolve inflammation, and maintain alveolar bone. Further research is needed to get insights into the mechanisms behind the relationships among SCFA receptors and LCFA receptors in innate immune cells. This will allow for a better understanding of host-commensal crosstalk to prevent pathogenic transformation of the oral microbiome.

## Data Availability Statement

The original contributions presented in the study are included in the article/Supplementary Material, further inquiries can be directed to the corresponding author.

## Ethics Statement

The studies involving human participants were reviewed and approved by Institutional Review Board of the Harvard Longwood Medical Area (IRB19-1697). The patients/participants provided their written informed consent to participate in this study.

## Author Contributions

MW designed the study, performed experiments, acquired and analyzed data (flow cytometry and questionnaire data), and drafted the manuscript. SB contributed to study design and data analysis (flow cytometry) and reviewed the manuscript. PB analyzed data (microarray data) and reviewed the manuscript. KH contributed to study design and data acquisition (flow cytometry) and reviewed the manuscript. CS designed the study and contributed to data analysis and manuscript preparation. All authors contributed to the article and approved the submitted version.

## Funding

This work was supported by the NIH/NIDCR through USPHS grants R00-DE024575 (CS) and the Harvard School of Dental Medicine startup fund to the Sima Lab.

## Conflict of Interest

The authors declare that the research was conducted in the absence of any commercial or financial relationships that could be construed as a potential conflict of interest.

## Publisher's Note

All claims expressed in this article are solely those of the authors and do not necessarily represent those of their affiliated organizations, or those of the publisher, the editors and the reviewers. Any product that may be evaluated in this article, or claim that may be made by its manufacturer, is not guaranteed or endorsed by the publisher.
