# Supplementary material for: Oral Neutrophil Free Fatty Acid Receptors Expression May Link Oral Host and Microbiome Lipid Metabolism
Source: Front Oral Health. 2022 Mar 7;3:821326. doi: 10.3389/froh.2022.821326 (PMC8937037; doi:10.3389/froh.2022.821326)
Supplement: Supplementary file 1 [file Data_Sheet_1.PDF]

Table S1. Study questionnaire

Gender: M [] F[] Age: Study ID:	Yes	No	Notes
1. Have you been diagnosed with periodontitis (gum disease)?	[]	[]	If yes, specify year of diagnosis: Has it been treated? Yes [] No [] N/A []
2. Do you have any oral conditions other than caries (tooth decay) and periodontitis?	[]	[]	If yes, please specify here. Oral conditions:
3. Do you have any non-oral chronic conditions?	[]	[]	If yes, please specify here. Conditions:
4. Are you taking any medications?	[]	[]	If yes, specify here. Medications:
5. Are you taking baby aspirin (81 mg)?	[]	[]	If yes, please specify when started. Aspirin since:
6. Are you taking omega-3 supplements?	[]	[]	If yes, specify dose here. Omega-3 Dose:
7. Do you eat fish every week?	[]	[]	If yes, specify number of servings. Fish Servings per Week:
8. Is your body mass index (BMI) over 25?	[]	[]	Please specify your BMI (weight/height2) BMI:
9. Female only: Are you pregnant?	[]	[]	If yes, please specify trimester. Trimester of pregnancy:
10. Have you had a physical exam in the past year?	[]	[]	Please specify abnormal findings if any. Findings:

Table S2. Subject characteristics

	Males	Females
**Total Number (n)**	10	10
**Ethnicity (n)**		
White	7	7
Black	0	0
Asian	3	3
**Age (mean±SD)**	39.3±18.17	34.2±13.1
**BMI (mean±SD)**	23.6±2.7	21.5±2.1
**Fish Servings (mean±SD)**	1.1±1.6	1.4±1.1
**Low Dose Aspirin (n)**	0	0
**Omega-3 Supplement**	1	0
**Hypertension (n)**	1	0
**Diabetes Mellitus (n)**	0	0
